# First-person view of one’s body in immersive virtual reality: Influence on episodic memory

**DOI:** 10.1371/journal.pone.0197763

**Published:** 2019-03-07

**Authors:** Lucie Bréchet, Robin Mange, Bruno Herbelin, Quentin Theillaud, Baptiste Gauthier, Andrea Serino, Olaf Blanke

**Affiliations:** 1 Laboratory of Cognitive Neuroscience, Brain Mind Institute, School of Life Sciences, Swiss Federal Institute of Technology (EPFL), Campus Biotech, Geneva, Switzerland; 2 Center for Neuroprosthetics, Swiss Federal Institute of Technology (EPFL), Campus Biotech, Geneva, Switzerland; 3 Department of Neurology, Geneva University Hospital, Geneva, Switzerland; Anglia Ruskin University, UNITED KINGDOM

## Abstract

Episodic memories (EMs) are recollections of contextually rich and personally relevant past events. EM has been linked to the sense of self, allowing one to mentally travel back in subjective time and re-experience past events. However, the sense of self has recently been linked to online multisensory processing and bodily self-consciousness (BSC). It is currently unknown whether EM depends on BSC mechanisms. Here, we used a new immersive virtual reality (VR) system that maintained the perceptual richness of life episodes and fully controlled the experimental stimuli during encoding and retrieval, including the participant’s body. Our data reveal a classical EM finding, which shows that memory for complex real-life like scenes decays over time. However, here we also report a novel finding that delayed retrieval performance can be enhanced when participants view their body as part of the virtual scene during encoding. This body effect was not observed when no virtual body or a moving control object was shown, thereby linking the sense of self, and BSC in particular, to EMs. The present VR methodology and the present behavioral findings will enable to study key aspects of EM in healthy participants and may be especially beneficial for the restoration of self-relevant memories in future experiments.

## Introduction

A defining feature of episodic memory (EM) is the capacity to provide information about the content of our conscious personal experiences of “when” and “where” events occurred as well as “what” happened [1,2]. Previous studies defined EM as the recall of contextually rich and personally relevant past events that are associated with specific sensory-perceptual and cognitive-emotional details [3–10]. EM has been distinguished from semantic memory, the latter being associated with general self-knowledge and the recall of personal facts that are independent of re-experiencing specific past events [11–17].

In a series of seminal papers, Endel Tulving highlighted the subjective dimension of EM associated with the re-experiencing of specific past events by pointing out the importance of the sense of self and introducing his influential notion of autonoetic consciousness. He argued that autonoetic consciousness is of fundamental relevance to EM, allowing one to mentally travel back in subjective time and recollect one’s previous experiences [2,18–20]. Tulving distinguished autonoetic consciousness from noetic consciousness, linking the latter to semantic memory and to knowing about (rather than re-experiencing) specific past events. Others extended Tulving’s notion of EM and proposed that it is contributing to the sense of self across time [10,12,21–25] and developed behavioral tasks such as mental time travel [26–31].

Although, several other cognitive domains have been proposed to contribute to the sense of self (i.e. language, mental imagery, facial self-recognition [32–35]), recent research has highlighted the importance of non-cognitive multisensory and sensorimotor contributions to the sense of self. This novel theoretical and experimental approach is based on behavioral [36,37], neuroimaging [38–40] and clinical data [39,41] and involves the processing and integration of different bodily stimuli to the sense of self: bodily self-consciousness (BSC) (for review see [42,43]). BSC includes conscious experiences such as self-identification and self-location [36,37,44,45], as well as the first-person perspective [39,46,47]. This work was based on clinical observations in neurological patients with so-called out-of-body experiences characterized by changes in the sense of self, in particular of the experienced self-location and perspective from an embodied first-person perspective to a third-person perspective [39,41] and has been able to induce milder, but comparable, states in healthy participants using virtual reality (VR) technology to provide multisensory stimulation [36,39,47].

Given the link of BSC with subjective experience and previous claims that subjective re-experiencing of specific past events is a fundamental component of EM [2,18], we argue that multisensory bodily processing may not only be of relevance for BSC, but also for consciousness concerning past events. Recent findings have shown that BSC impacts several perceptual and cognitive functions such as tactile perception [48,49], pain perception [50,51], visual perception [52–54], as well as egocentric cognitive processes [55]. Concerning EM, St. Jacques et al. [56] used a novel camera technology to examine the differences in self-projection (i.e. the capacity to re-experience the personal past and to mentally infer another person’s perspective) and found that the ventral–dorsal subregions of the anterior midline are functionally dissociable and may differentially contribute to self-projection when comparing self versus other. Bergouignan et al. [57] reported that recall of items and hippocampal activity during the encoding of episodic events is modulated by the visual perspective from where the event was viewed during encoding and St. Jacques et al. [58] showed that first- versus third-person perspective during retrieval modulated recall of autobiographical events and associated this with medial and lateral parietal activations. Together, these findings revealed that retrieval-induced forgetting is enhanced by third-person, but not first-person perspective. Therefore, these studies suggest that encoding of EM requires the natural co-perception of one’s body and the extrapersonal world, which is perceived from the first-person perspective. As such, we here predicted that bodily multisensory processing, that has been described to modulate BSC, would interfere with EM processes.

Traditionally, behavioral and neuroimaging EM studies rely on questionnaires, verbal reports, interviews, or mental imagery and predominantly investigated memory retrieval by using a variety of stimuli and procedures such as cue words and pictures [58–63]. For example, important research relied on interviews with the participants [61,64] on personalized lists of significant life events of participants [9,30,65–67], and employed different procedures asking participants to re-experience particular life episodes [59,62,63,68,69]. This differs from research investigating verbal memory through encoding and recall of word lists [70–73] or testing spatial memory with figures, spatial paths, or other visuospatial materials [74–76] (for which it is much easier to fully control encoding and retrieval). Beyond the use of controlled images, short video clips or words in EM studies [4,77], an important line of neuroscientific EM work has used novel approaches employing stimuli from real world encounters, outside the laboratory. For example, Cabeza et al. [65] created a campus tour paradigm and tested EM retrieval by using digital photos taken from the tour. Similarly, Schacter et al. [78] introduced a museum tour paradigm, which was used to study the reactivation-induced updating in memory for events experienced during the tour. Thus, during encoding, participants went on an audio-guided museum tour, while wearing a camera which automatically took photos some of which were selected to test EM (see also [56]). Vogel and Schwabe [79] also used pictures, which were taken automatically and continuously by a camera during a 2-hour walk through a zoo for testing EM, comparing events represented by pictures from their own zoo tour with those of others. Several EM research groups have relied on advances in video technology and VR during encoding and retrieval of information (i.e. spatial navigation [80,81]; social interactions [82,83]). Participants were seated in front of a computer screen showing a virtual environment and asked to navigate in such environments using a joystick (encoding) and later asked to recall selected items from the environment (retrieval). These computer-based VR studies suggest that both interactions with the environment during encoding or retrieval influence memory performance. Compared to passive participation, several VR studies showed better learning performances across free recall trials and recognition tasks [80,84–86]. Plancher et al. [87] suggested that interactions with the naturalistic environment created with VR enhanced spatial memory. However, despite these important achievements, these virtual environments were mostly using non-immersive VR systems, did not employ real life like virtual scenes, and did not use VR technology that allows integrating the participants’ body (and hence multisensory bodily stimulation) for the tested virtual life episodes. In the present experiments, we took advantage of a recently developed immersive VR system, which allows us to preserve the perceptual richness of life episodes, to fully control the experimental stimuli during encoding and retrieval, and to integrate and manipulate multisensory information of our participant’s body in an online fashion. Unlike in traditional, laboratory-based studies, here we claim that particularly the presence of one’s own physical body plays a crucial role in our experimental testing of EMs. Our paradigm approaches 3D real life episodes, but in a VR setting for which all items of the scene during encoding and retrieval are fully controlled. This VR technology allows us to examine the relation between “the bodily-self” and “the episodic-self”, particularly the subjective experience of mentally travelling back in time. The present experiments had one major technological and one major scientific goal: (1) develop and test real life-like memory in the laboratory with virtual episodes using immersive VR and (2) investigate whether multisensory bodily stimulations that have been shown to impact BSC, perception, and egocentric cognition modulates EM.

In the first experiment, we tested our immersive VR system and sought to address some of the experimental limitations of earlier EM studies, which either had limited control of actual autobiographical stimuli and events during encoding and only examined the stage of EM retrieval [5,60,67,88] or controlled EM encoding, but without the immersion into the original scenes during EM retrieval [9,57,65]. The main aim of our first experiment was to validate our novel VR paradigm in order to study EM in a more naturalistic setting. We further tested EM performance and confidence for immersive three-dimensional (3D) VR scenes at two different time points and for different number of items (that changed between both sessions), we predicted memory decreases depending on delay and on the number of items changed. Numerous behavioral cognitive studies have observed dissociations between memory accuracy and memory confidence [89–95]. For example, Talarico & Rubin [89] showed that the objective accuracy for events of September 11^th^, 2001 did not differ from accuracy in every-day events. However, the subjective feeling of remembering was enhanced for the highly arousing EMs compared to everyday-like EMs. Likewise, Sharot & Yonelinas [91] found that emotional photographs were remembered with a greater subjective sense of recollection, yet the objective memory performance between emotional and neutral photos did not differ. Similar to the prior investigations examining the effect of emotional memories on subjective confidence, we thus sought to investigate the impact of multisensory bodily cues on subjective confidence.

In the second experiment, we investigated the main scientific hypothesis of the present experiments and tested the potential link between multisensory own body signals, that are fundamental for BSC and EM. Vision and proprioception are sensory signals that are highly relevant for the brain in order to rapidly and continuously update the instantaneous representation of the body in space. Perceiving one’s body as part of a visual scene (for example a hand lying on a table) relies on i. visual, ii. proprioceptive, and iii. tactile cues. These signals are processed initially in different brain regions and subsequently integrated in multisensory brain regions [42,43]. Such multisensory body-related signals are not just relevant for hand perception, but also for BSC, including hand ownership (i.e. the feeling that this hand is mine), self-identification with the body, self-location (i.e. experiencing the self as being located in space), and the first-person perspective (i.e. experiencing the world from a spatial origin with a direction) [42,43,96]. We thus examined whether the presence of online and congruent multisensory cues from the participant’s body (i.e. the presence of one’s own physical body from the first-person viewpoint) impacts memory performance and confidence in the present VR paradigm, compared to an experimental condition where such online first-person bodily cues are absent. Based on BSC work that has shown that view of the body enhances perceptual and cognitive tasks [57,58] and based on the fact that during memory encoding the body is in most instances co-perceived with the other elements of the scene, we predicted that the presence of a body during encoding would enhance memory performance. Finally, we performed a third (control) experiment in order to test whether the effect of multisensory bodily stimulation that we observed in the second experiment is specific to multisensory bodily cues.

## Methods

### Participants

A total of 79 participants with normal or corrected to normal vision were recruited to the study. None of the participants indicated neurological or psychiatric deficits and all participants were right-handed. In experiment 1, 16 participants (M = 23.7 years, SEM = 0.7 years, 8 female) participated in the immediate recognition group and 15 right-handed participants (M = 23.4, SEM = 0.8, 7 female) participated in the one-hour delayed recognition group. In experiment 2, 16 participants (M = 26.8 years, SEM = 0.6, 4 female) participated in the immediate recognition group and 16 participants (M = 24.5 years, SEM = 1.1, 8 female) participated in the one-hour delayed recognition group. In experiment 3, 16 right-handed participants (M = 25.4, SD = 3.7, 7 female) participated in one-hour delayed group. Sample size was derived from power analysis of previous BSC studies [97,98] and previous studies on mental time travel [28,30]. According to these studies, our target effect size was 0.6 for the present experiments. The study was approved by the local ethical committee (IRB of Geneva University) and all three experiments were conducted in conformity with the Declaration of Helsinki. Written and signed informed consents were obtained from all participants.

### Virtual reality technology

Our VR technology uses a spherical capturing and recording system and an immersive setup for first-person perspective (1PP) replay of the recorded real environments. For recording a scene, 14 cameras (GoPro Hero4) are assembled on a spherical rig (360hero 3DH3PRO14H) and linked to 4 pairs of binaural microphones (3DIO Omni Binaural Microphone) to cover the entire sphere of perception around a viewpoint (360° horizontally and vertically, stereoscopic vision, binaural panoramic audio). A custom software (Reality Substitution Machine, RealiSM, http://lnco.epfl.ch/realism) then aggregates all data into a single high-resolution panoramic audiovisual computer format (equivalent to more than 4 stereoscopic full HD movies). A head-mounted display (HMD, Oculus Rift DK2; 900x1080 per eye, FOV ~105° Vertical, 95° Horizontal) was used to immerse participants into the recording and sound was administered with noise-cancelling headphones (BOSE QC15). Furthermore, the HMD was coupled with a stereoscopic depth camera (Duo3D MLX, 752x480 at 56Hz) mounted on its front face to capture participants’ bodies from 1PP. The RealiSM software then augments the fully immersive environment with a realistic view from which participants could see their hands, trunk and legs from 1PP. As a result, participants experienced as if they would be physically present in the pre-recorded scenes and seeing oneself (not a 3D avatar). The software also allows integrating 3D virtual items seamlessly in the scene (experiment 3).

### Stimuli

Participants were immersed in three (experiment 1) or two (experiments 2 and 3) pre-recorded rooms via the HMD (see below). For the encoding session, 10 everyday-life items (e.g., coffee machine, pen, trash bin) were placed in each room. These real-life items created the natural context of episodic memory at both encoding and retrieval. During retrieval, rooms remained either exactly the same as during encoding (i.e. the same 10 real-life items were again presented at the same places in the previously visited rooms) or some of the items (i.e. 1, 2 or 3 items) were replaced by new items that were not previously seen in any of the scenes. Each room included different set of items in order to keep the same level of novelty and thus avoid any facilitation on the following recognition task.

### Paradigm

**Fig 1:**
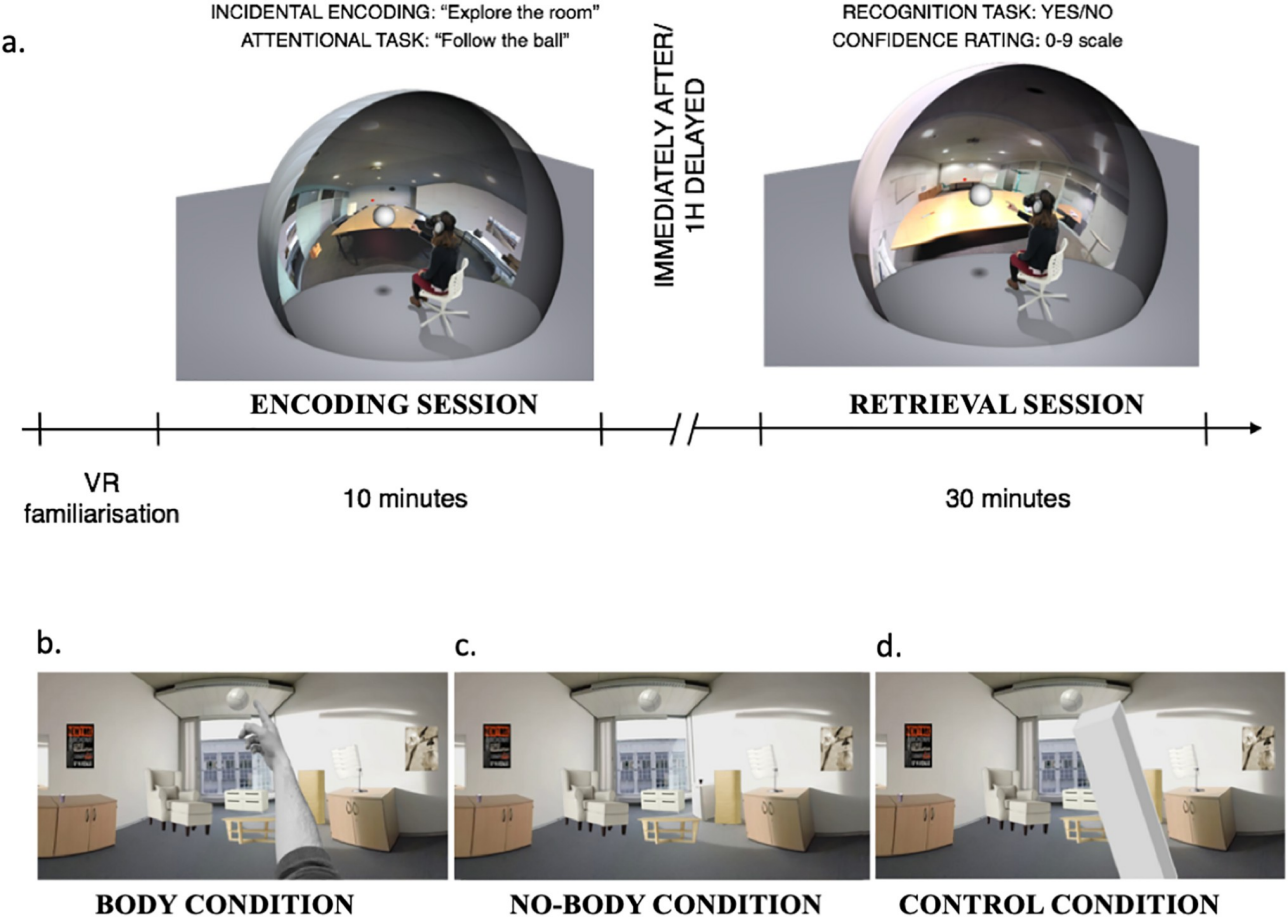
Experimental procedure and 3D scenes. After a period of familiarization with the immersive VR setup, participants performed the encoding session (10 minutes) during which they were exposed to different life-like 3D scenes (Fig 1a). Scenes were characterized by a room that contained different items (table, photocopy machine, pen, etc.). In experiment 1, one group of participants performed the retrieval session (30 minutes) immediately after the encoding session or after a one hour delay (see main text for further detail). Figs 1b-d shows the different conditions during the encoding session that we used in experiments 1–3 (the retrieval session was the same across all experiments). Thus, participants always saw the same 3D scenes on the head-mounted display, but the body of the participant was either not seen at all (Fig 1b; body condition), seen as part of the 3D scene (Fig 1c; no-body condition), or instead of the body a control object was seen (Fig 1d; control condition).

Each of the three experiments consisted of two sessions, an incidental encoding period (session 1) followed by an immediate (group 1) or one-hour delayed (group 2) surprise recognition task (session 2). We studied incidental encoding (i.e. participants were unexpectedly given a recognition test) in order to examine which of the every-day items would participants remember without expecting the recognition test. In all three experiments, participants were not informed that we would later test their memory for the stimuli encountered during the encoding session. Before the two experimental sessions, participants were seated on a chair and asked to put on the HMD and headphones. Before the two experimental sessions, participants were asked to put on the HMD and headphones. In order to familiarize with the VR technology, each participant was immersed into an outside scene for 5 minutes, which we have recorded in a park close to the Lake of Geneva. We specifically asked our participants to remain seated, turn and look around and to explore the scene as if they were sitting on a bench in the park. The lake scene consisted of a blue sky, there were trees behind them, and there was a view on the lake in front of them overlooking the French Alps on the other sides of the lake). Paradigm and testing sequence are depicted in Fig 1a.

### Encoding session

During the encoding session, to assure that participants explored the entire room and to monitor their attention within the different 3D scenes (i.e. the different rooms), participants were instructed to freely explore each virtual room. Moreover, we programmed a virtual ball that appeared in each of the three rooms and was moving within the rooms for a duration of 30 seconds and covered all sections of the virtual room. Participants were asked to fixate the virtual ball and to follow its movements through the virtual room. In total, the target items in the scenes, which were questioned during the retrieval session appeared at 6 different positions in each room. After the ball stopped moving, participants freely explored each room for another 30 seconds.

The procedure in experiment 2 was identical. However, in order to test the effect of viewing one’s own body during encoding we asked participants to follow the trajectory of the ball and to point at the moving ball with their hand and finger. The main manipulation consisted in showing the participant’s physical body (body condition) or not (no-body condition). This was accomplished with the use of the stereoscopic depths cameras to capture in the participant’s body and by turning them on in the body and off in the no-body condition. The participant’s body was inserted in real-time in the virtual room and shown from the habitual visual first-person viewpoint. In the body condition, participants saw their physical hand, the trunk, and their legs (i.e. the stereoscopic depths cameras were turned on) in the HMD and as part of the virtual 3D scene (Fig 1b). In the no-body condition, the virtual 3D scene was identical except that the participant’s body was missing (i.e. the stereoscopic depths cameras were turned off) (Fig 1c). The order of presentation of the body and no-body condition was counterbalanced between participants. In experiment 2 each participant explored two rooms (i.e. with 3 rooms as in experiment 1 the experiment would have been too long).

In experiment 3, participants were also asked to follow the movement of the ball appearing in each room (by physically pointing at it with their hand and finger). Yet in the object condition they were shown a non-bodily control object, instead of their own physical body (Fig 1d). The no-object condition was the same as the no-body condition in experiment 2. The presentation of the object and no-object condition was counterbalanced between participants. No explicit instructions to memorize items of visited rooms were provided. In experiment 3, each participant explored two rooms (i.e. to keep conditions comparable with respect to experiment 2).

#### Retrieval session

During the retrieval session, which was the same for all three experiments (i.e. no-body or control object was shown), participants were informed that they would be immersed in the same rooms again. They performed a total of three blocks of 40 trials (each lasting 10 seconds). Within the three blocks of 40 trials, we presented 10 trials, which were exactly the same as during the original encoding session (i.e. including the same 10 items). The remaining 30 trials were different and had either 1, 2 or 3 new items replacing the respective number of items shown during the encoding session. The blocks and individual trials in each block were presented in a randomized order. Participants were free to re-explore the virtual scenes for 10 seconds, after which they were asked two questions that were shown on the HMD. First, participants performed a two-alternative forced choice task (yes/no) whether the virtual scene shown during the retrieval session corresponded to the virtual scene during encoding (recognition task) (“Is the scene exactly the same as when you first saw it?”). Participants indicated their response with a wireless computer mouse. Second, participants were asked how confident they were about their answer (via a rating scale projected in the HDM; range from 0 (low) to 9 (high confidence)).

### Statistical analysis

In experiment 1, an independent samples t-test for hit rate and false alarm rate was applied to test whether EM performance differed depending on delay (i.e. immediate x one-hour delayed). Independent sample t-test were further used to analyze whether the hit rate and false alarm for EM confidence ratings differed depending on delay. A mixed analyses of variance (ANOVA) with the number of items changed (i.e. 1 item, 2 items or 3 items) and delay (i.e. immediate x one-hour delayed) was performed. Further, a 2 x 3 mixed ANOVA was run to understand the effects of delay (i.e. immediate x one-hour delayed) and number of items changed in a room (i.e. 1 item, 2 items, 3 items) for the EM confidence for the false alarm rates. Where appropriate, Greenhouse-Geisser corrections of degrees of freedom were used. Significant ANOVA effects were explored by post-hoc tests using Bonferroni correction. The significance level was set to alpha 0.05.

In experiment 2, we performed a mixed analysis of variance (ANOVA) with delay (i.e. immediate x one-hour delayed) and body (i.e. body x no-body) as categorical factors on EM performance for hit rate and 2 (i.e. immediate x one-hour delayed) x 2 (i.e. body x no-body) x 3 (i.e. 1 item, 2 items or 3 items) mixed analysis of variance (ANOVA) for false alarms. Where appropriate, Greenhouse-Geisser corrections of degrees of freedom were used. Significant ANOVA effects were explored by post-hoc tests using Bonferroni correction. The significance level was set to alpha 0.05.

For EM confidence, a 2 x 2 x 3 mixed ANOVA was performed with delay (i.e. immediate x one-hour delayed), body (i.e. body x no-body) and number of items changed (i.e. 1 item, 2 items or 3 items) as categorical factors. Where appropriate, Greenhouse-Geisser corrections of degrees of freedom were used. Significant ANOVA effects were explored by post-hoc tests using Bonferroni correction. The significance level was set to alpha 0.05.

In experiment 3, an independent samples t-test was applied to test whether EM performance differed in the object versus no-object condition. This was done for hit rate and for false alarm rate. An independent sample t-test was also used to examine whether EM confidence false alarm differed in the object versus no-object condition. A mixed analyses of variance (ANOVA) with the number of items changed (i.e. 1 item, 2 items or 3 items) and object (i.e. object x no-object) was performed. Similarly, a 2 x 3 mixed ANOVA was run to understand the effects of object (i.e. object x no-object) and number of items changed in a room (i.e. 1 item, 2 items, 3 items) for the EM confidence for the false alarm rates. Where appropriate, Greenhouse-Geisser corrections of degrees of freedom were used. Significant ANOVA effects were explored by post-hoc tests using Bonferroni correction. The significance level was set to alpha 0.05.

## Results

### Experiment 1 (Immediate versus one-hour delayed group)

**Fig 2:**
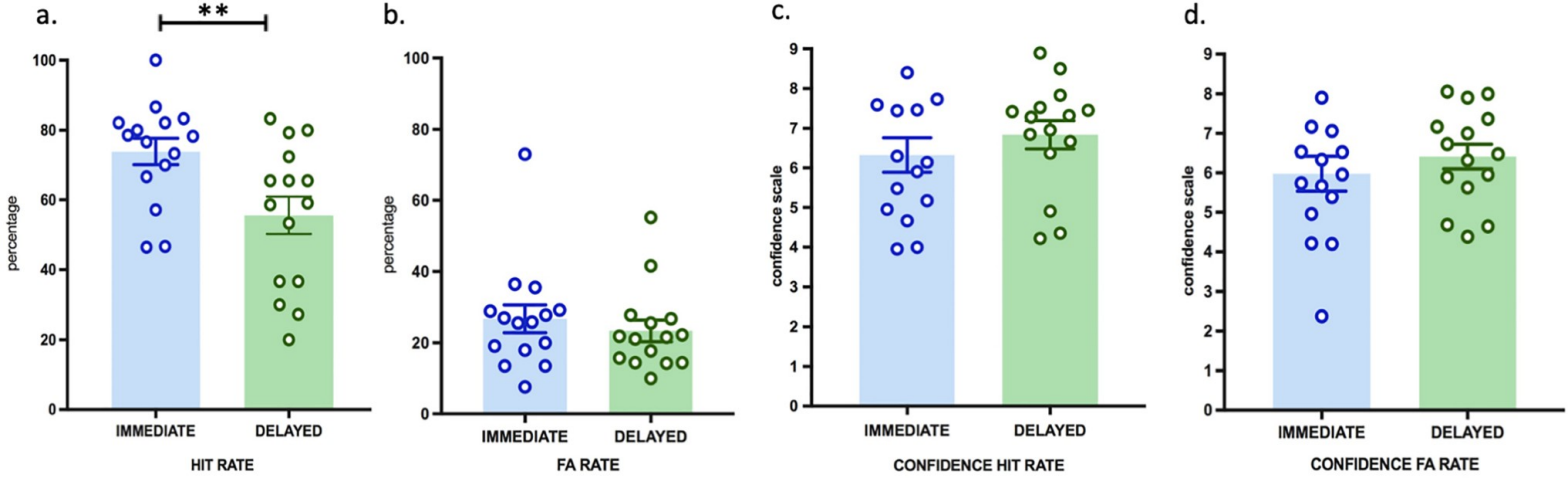
EM performance in experiment 1 (immediate versus one-hour delay condition). EM performance (hit rate, false alarm rates) and subjective confidence ratings are indicated in percentage + SEM. (**) P < 0.01; (*) P < 0.05. Fig 2a. Hit Rate; Fig 2b. False Alarm Rate; Fig 2c. Confidence ratings (Hits); Fig 2d. Confidence ratings (False alarms). Blue color represents the immediate condition. Green color represents the delayed condition.

Participants in the delay group showed a significant decline in performance compared to the immediate memory recognition group. Mean hit rate was significantly lower in the delay group (M = 55.5, SEM = 5.3) than in the immediate group (M = 73.1, SEM = 3.6) (t (29) = 2.7, p = 0.01) (Fig 2a). False alarm rates did not differ between both groups (immediate group: M = 31.4, SEM = 5.8; delay group: M = 23.3, SEM = 3.0; t (29) = 1.1, p = 0.2) (Fig 2b). These data show that participants recognized 3D scenic events better when tested immediately after the exposure than when tested with a delay of one hour, without any effect of delay on false recognitions.

Confidence ratings for hits in the immediate group (M = 6.2, SEM = 1.6) were not significantly different from those in the delay group (M = 6.8, SEM = 1.3) (t (29) = 1.09, p = 0.2) (Fig 2c). The same was found for false alarms confidence that did not differ between the immediate group (M = 5.8, SEM = 0.4) and delay group (M = 6.4, SEM = 1.2) (t (29) = 0.7, p = 0.3) (Fig 2d). Thus, despite changes in recognition, confidence did not differ depending on delay.

**Fig 3:**
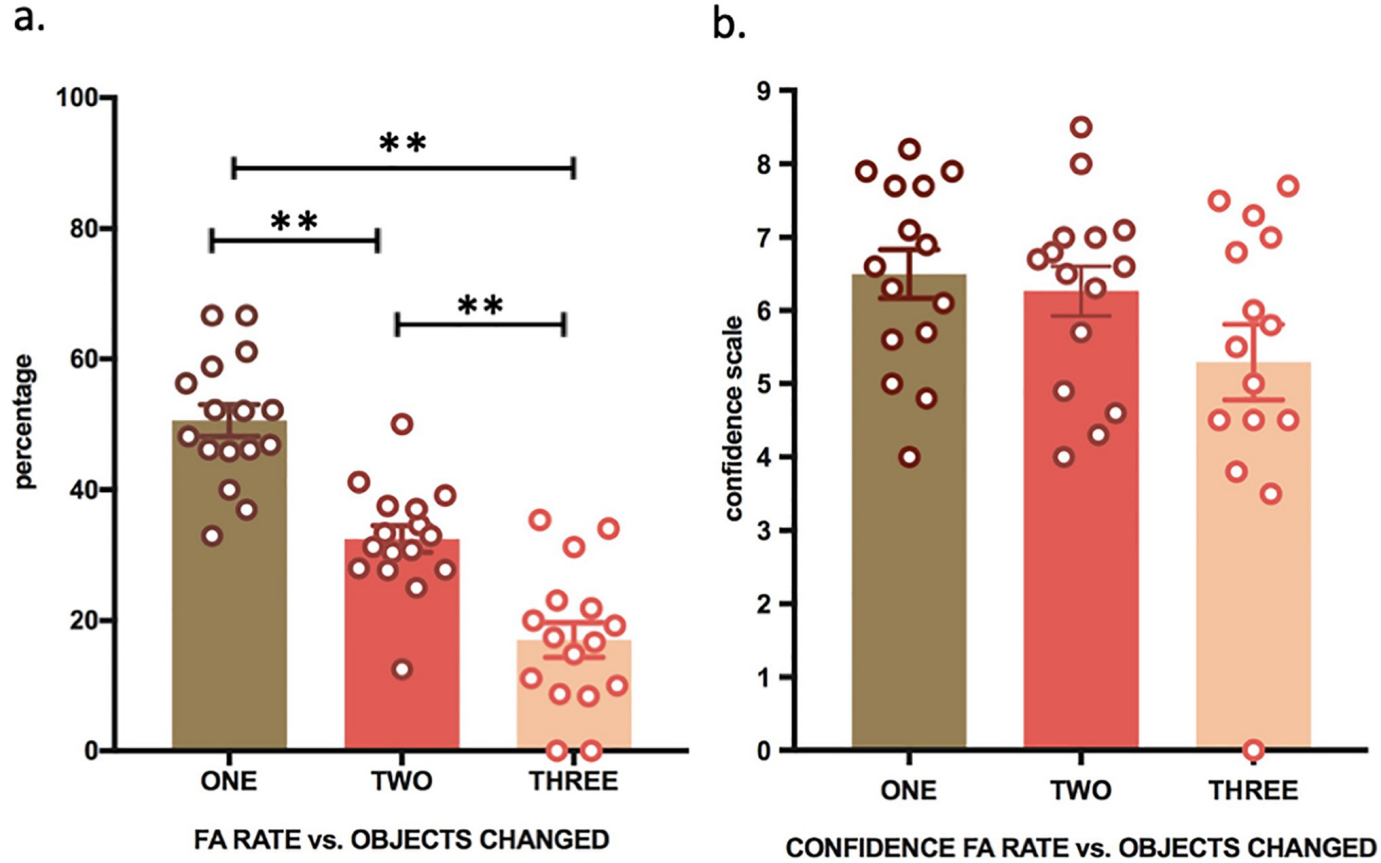
False alarms depend on number of items changed (experiment 1). EM performance (false alarms) is indicated in percentage + SEM. (**) P < 0.01; (*) P < 0.05. Fig 3a. False Alarm versus Number of Items changed (i.e., 1 item, 2 items, 3 items); Fig 3b. Confidence Rate for False Alarm versus Number of Items changed (i.e., 1 item, 2 items, 3 items).

We next examined whether performance in the present task depended on the number of items changed within each immersive 3D scene. This analysis was conducted on the false alarm rate (as no items changed for hits, by definition). As predicted, analysis revealed a significant main effect for the number of items changed (F (2, 58) = 52.85, p < 0.0005, partial η^2^ = 0.64) (Fig 3a). Pairwise comparisons were performed for statistically significant main effects and revealed that participants made progressively fewer false alarms with increasing number of items (all p-values < 0.0005).

There was also a statistically significant main effect for the number of items changed (F (1.121, 32.519) = 4.163, p = 0.02, partial η^2^ = 0.12) (Fig 3b), revealing that participants were progressively more confident in their performance with increasing number of items that were changed between both sessions. These data show that participants made more recognition errors and were less confident in conditions in which less items were changed between encoding and retrieval.

### Experiment 2 (Body versus no-body condition)

**Fig 4:**
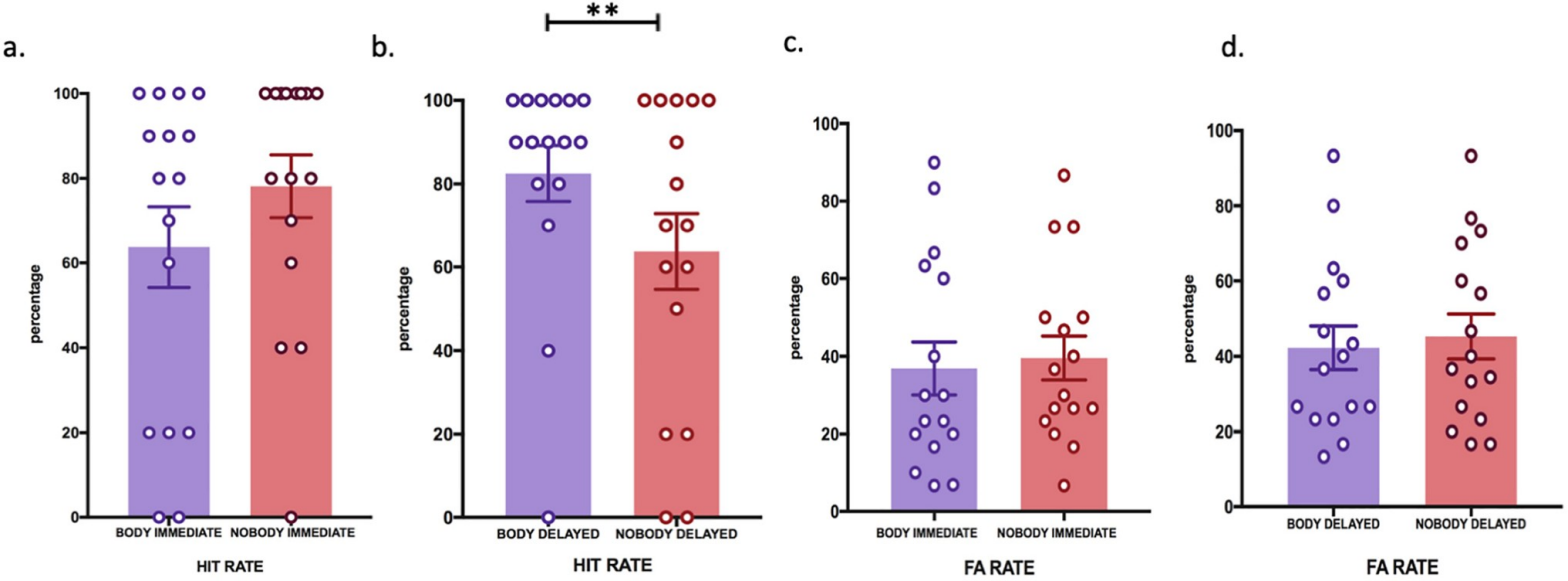
Body view enhances recognition (experiment 2). Immediate versus one-hour delay EM performance is indicated in percentage + SEM is indicated. (**) P < 0.01; (*) P < 0.05. Fig 4a. Hit Rate in immediate body (in purple color) versus immediate nobody (in pink color) condition; Fig 4b. Hit Rate in delayed body (in purple color) versus delayed nobody (in pink color) condition; Fig 4c. False Alarm Rate in immediate body (in purple color) versus immediate nobody (in pink color) condition; Fig 4d. False Alarm Rate in delayed body (in purple color) versus delayed nobody (in pink color) condition.

Data for hit rates showed a significant two-way interaction between the time of retrieval and body conditions (F (1,30) = 7.44, p = 0.01, partial η^2^ = .19). Post-hoc testing revealed that this effect was explained by a higher hit rate in the body, which was found specifically in the delay condition (body: M = 82.5, SEM = 8.2; no-body condition: M = 63.7, SEM = 8.2; *t* (15) = 2.51, *p* = 0.02), but not in the immediate condition (Fig 4a and 4b). These data show that recognition of immersive 3D scenes, that also include the first-person view of the participant’s body, mimicking real-life experience is modulated and enhanced with respect to the same scenes without such a bodily view. The 2 x 2 x 3 ANOVA for false alarms revealed a significant main effect for the number of items changed, F (1.173, 17.591) = 20.921, *p* < 0.001 (S1 Fig). The other main effects and interactions were not significant (Fig 4c and 4d).

Confidence ratings for hits did not reveal any differences between the time of retrieval and body conditions. The 2 x 2 x 3 ANOVA for confidence judgements in false alarms revealed a significant main effect for the number of items changed, F (2, 30) = 8.38, *p* = 0.001. The other main effects and the interactions were not significant. Thus, despite changes in recognition, confidence did not differ depending on time of retrieval or body conditions.

### Experiment 3 (object versus no-object condition)

**Fig 5:**
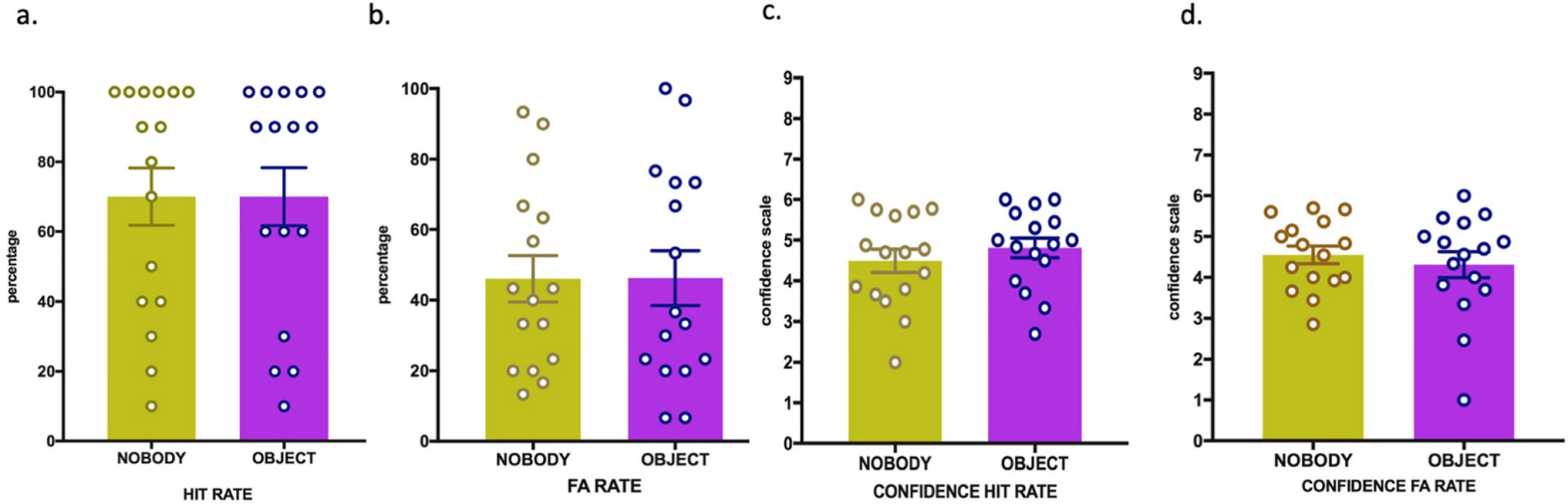
No difference between object and no-object view (experiment 3). One-hour delay EM performance is indicated in percentage + SEM is indicated. (**) P < 0.01; (*) P < 0.05. Fig 5a. Hit Rate for object versus no-object condition; Fig 5b. False Alarm Rate for object versus no-object condition; Fig 5c. Confidence ratings (Hits); Fig 5d. Confidence ratings (False alarms).

There was no significant difference in hit rates for participants in the object condition (M = 70.0, SEM = 8.3) compared to the no-object condition (M = 70.0, SEM = 8.2) (*t* (15) = 0.00, *p* = 1.00) (Fig 5a). Similarly, false alarm rates did not differ between conditions (object condition: M = 46.2, SEM = 7.7; no-object condition: M = 46.0, SEM = 6.5; *t* (15) = -0.05, *p* = 0.96) (Fig 5b). These data show that recognition of immersive 3D scenes, where a non-bodily object, instead one’s own body, is visible from the first-person view, does not modulate performance in the present task with respect to the same scenes without rectangular control object.

Confidence for hits in the object condition (M = 4.8, SEM = 0.2) was not significantly different from the no-object condition (M = 4.4, SEM = 0.2) (Fig 5c). Confidence for false alarm also did not differ between conditions (object condition: M = 4.3, SEM = 0.3; no-object condition: M = 4.5, SEM = 0.2) (Fig 5d).

Further, we examined whether memory performance depended on number of items changed and the object vs. no-object condition. The analysis revealed a significant main effect for the number of items changed for the false alarm rate (F (2, 30) = 7.79, p < 0.0005, partial η^2^ = 0.34). Post-hoc analysis revealed a statistically significant change from 1 item to 3 items (p = 0.01; Bonferroni corrected). No statistically significant two-way interaction was found between the object conditions and number of items changed. There was no significant difference between the no-object and object conditions.

We also tested whether the confidence in the performance accuracy depended on the number of changed items within each scene and the object condition. The main effect showed a statistically significant difference for the number of items changed (F (2, 30) = 3.42, p = 0.04, partial η^2^ = 0.18). No statistically significant two-way interaction was found between the confidence ratings for the object conditions and number of items changed.

## Discussion

The present study allows us to draw three major conclusions. First, the present VR setup permits to measure recognition memory for 3D scenes that are immersive, rich in contextual detail, and that further integrates the moving body of the participant in online fashion. Our VR setup, thus, approaches real-life experiences in controlled laboratory conditions. Moreover, the present VR setup allowed us to project the same 3D virtual scenes during the encoding and retrieval sessions, providing us arguably with a level of experimental control that is comparable to examinations of classical word/picture-based recognition paradigms [99–102], which are typically used to study memory. Second, applying this new setup we report that recognition memory for the tested VR scenes depends on the delay and on the number of changed elements between encoding and retrieval, comparable to findings for verbal and visual-spatial memory. Third, we show that viewing one’s body as part of the virtual scene during encoding enhances delayed retrieval. This body effect was not observed when no virtual body was shown or when a moving control object (instead of the virtual body) was shown, suggesting that embodied views lead to body-related performance changes, as reported in studies investigating BSC.

### An experimental VR setup that controls real-life like episodes during encoding and retrieval

Most prior laboratory-based EM studies used cue words or images to trigger memory retrieval and mental time travel to the past in a controlled fashion [5,6,12,31,60,80,87,103–105]. However, these studies controlled only for memory retrieval but not for memory encoding [4]. Contrary to these previous studies, we exposed our participants to rich and immersive real-life scenes without the need for explicit mental time travel. Unlike earlier computer-based scenarios, we also did not present participants with artificial scenarios (simulated events in 3D), but immersed them into 360° video recordings of everyday real-life scenes that we digitalized for the encoding and retrieval sessions. Using the present naturalistic and controlled VR setup, we ensured that our participants experienced virtual 3D scenes with congruent multisensory bodily information (visual, motor, vestibular); these approach real-life experience as compared to classical virtual computer game tasks that have been used for EM investigations in the past [106,107]. Thus, the present VR technology and future improvements of it will open new possibilities for conducting episodic memory research under ecologically valid experimentation in the laboratory by providing not only the ability to precisely design all stimulus aspects, but also to replay fully controlled sequences of real-life events.

### Delay and number of changed items modulates recognition memory performance

Our data reveal two classical episodic memory findings. Recognition memory for real-life like scenes decays with delay and improves depending on the number of items that were changed between encoding and retrieval. Previous EM research is compatible with these findings, but has not been able to test or quantify this directly. By definition, memories of each individual differ and cannot be reproduced across participants and studies. As such, our VR approach allows for more control of the environment, in particular the use of the exact same context for both the encoding and the retrieval phase as well as a digital method to manipulate and control the 3D stimulus material. Combining immersive VR with memory research thus allowed us to get both high control and reproducibility, while allowing to test real-life like scenes and events as compared to standard retrieval tasks. Specifically, while associative recognition memory for words or pictures [108–110] and EM [94,95,111] has been tested for different memory delays, previous VR-based paradigms, investigating the formation of episodic memory of life-like events, mostly tested immediate memory performance [81,82,112,113] (but see [114]). The present findings can be compared with classical memory findings for verbal and pictorial material where increasing delays increases forgetting [18,90,91,115,116] and with spatial memory work, where active navigation reduces forgetting as compared to passive viewing [80,84,85,87]. Thus, although we only tested short delays (i.e. one hour), our data show that participants remembered 3D scenes better when tested immediately after encoding as compared to delayed retrieval. Our second predicted finding that recognition memory was better when more items were changed between the encoding and retrieval is also compatible with classical findings concerning the recognition of visual changes when testing long-term memory for spatial scenes, complex figures (including faces), or short texts [117,118], further revealing the experimental validity of the present setup for research in episodic memory.

### Embodiment and episodic memory of life-like events

Besides reproducing classic memory effects, the present study also reveals a new finding, i.e. that memory is better when the body is visible at the encoding. Research on embodiment and BSC has used several VR paradigms and revealed the influence of multisensory and sensorimotor bodily input and has highlighted the importance of the view of the observer’s body [43]. Such research showed that BSC can be modulated by showing the body or body parts of the participant from different first-person viewpoints compared to showing no body at all. Moreover, this effect has been shown to be body-specific by demonstrating that different non-corporeal objects shown from the same position and viewpoint do not alter BSC [43]. Here, we extend this BSC principle to memory research by showing in experiment 2 that the recognition of 3D scenes that included within the first-person view also the participant’s body (as is characteristic of normal everyday perception) was modulated and significantly enhanced with respect to the same scenes without such a bodily view. This is compatible with previously reported effects for multisensory bodily perception [48,49] and BSC [37,39,44]. These BSC studies showed that visuo-tactile perception, as well as self-identification and self-location towards a seen human body or body part are enhanced when the body is shown in congruent position with respect to the participant’s body. Accordingly, we argue that the present body effect on the recognition memory of 3D scenes is comparable to similar effects in multisensory perception and BSC (i.e. for review see [43]) as well as a number of cognitive processes, where self-related bodily information is critical. For instance, viewing the body increases tactile perception [119], modulates interpersonal tactile responses [120,121], affects social cognition [122,123], and concept processing [55].

### The post-encoding modulation of EM performance at a delay due to the presence of bodily-self

It is of relevance to point out that the enhancement of EM performance in the body present condition was observed only in the one hour delayed retrieval session, but not when the retrieval followed immediately after the encoding session. Most events that people experience during their daily life will be forgotten. What determines which of the every-day experiences will be remembered? It has been well demonstrated that people tend to remember better those life-episodes, which are distinct and personally meaningful and, of importance for the present study, relate to the self or self-consciousness [21,24,28,30,124–126]. Additionally, the ability to discriminate among similar experiences is a crucial feature of episodic memory [127]. As such, the encoding while viewing one’s body may provide a better separation of memory traces than an encoding without a body as the multisensory integration of congruent signals from the body generates a more distinct target pattern to compare to the lures. Moreover, the typical delay for hippocampal consolidation processes starts at approximately one hour (for shorter delays it may rather relate to short-term memory, relying distinct mechanisms) [128–131]. Accordingly, we argue that enhanced self-relevance and recruitment of BSC-related processing in the present experiment (by viewing one’s body during encoding) improves the consolidation process of episodic memories, but not shorter-spanned memories. Similar delay-only effects have also reported during other manipulations. Thus, Sharot & Yonelinas [91] showed that emotion had no effect on recall when tested immediately after encoding, but only after a delay. Future work, including neuroimaging should investigate whether the modulation of delayed recall as described in the present experiments and those by previous authors rely on similar or distinct mechanisms. This testing may also include the investigation of additional BSC constraints (i.e. peripersonal space and embodiment [43,49,132]).

It could be argued that the enhanced EM performance of experiment 2 could relate to differences in the amount of visual information provided in both conditions (higher in the body versus the no-body condition) or higher salience or attention due to the additional inclusion of the tracked body in the body condition. First, we note that addition of the tracked body actually covers or hides parts of the virtual scene and may have thus incidentally hid some of the changed items and should thus rather decrease recognition memory. Yet, the opposite was observed in experiment 2. However, in order to formally investigate the potential role of differences due to vision or attention between conditions we compared, in experiment 3, the no-object condition with a condition in which participants viewed a non-bodily control object that was moving congruently with the participant’s body in real-time. Data from this experiment revealed no memory improvement in the object condition, arguing against a visual or attentional account and further corroborating our proposal that the present recognition enhancement is due to multisensory-motor bodily stimulation that has been shown to be crucial for BSC [36,42,49,133] and characteristic of normal everyday experience. These data also argue against the possibility that the present body effect on recognition memory can be generalized to an embodied object as the object condition did not induce any performance changes. Future EM studies should investigate other BSC aspects, such as peripersonal space, embodiment, and visual-proprioceptive alignment [43]. By revealing bodily effects in the present EM paradigm, we thus link BSC to EM, extending earlier memory work [57] that has focused on contributions of the first-person perspective in autobiographical memory or of vestibular processing on EM [134]. Finally, based on these data we argue that the brain mechanisms of BSC are linked to those of autonoetic consciousness that are of fundamental relevance to EM. Autonoetic consciousness is the ability to mentally travel back in subjective time and recollect one’s previous experiences [2,18–20] and the present data suggest that multisensory bodily processing during encoding and remembering are not only of relevance for the conscious bodily experiences of self-identification, self-location, and first-person perspective [36,37,39,44–47], but also autonoetic consciousness.

### Confidence and episodic memory

Does confidence mimic these changes in episodic memory performance? We report, as predicted, that confidence increased jointly with memory recognition improvements for conditions in which more items were changed. This finding is in line with several studies showing that confidence in everyday, non-arousing EM, measured by remember/know paradigms and recollection questionnaires, declines together with the objective memory performance [95,108,111]. However, our data also show that confidence levels dissociate from memory performance, as delay dependency and the view of one’s body (experiment 2) during encoding modulated recognition memory, but not confidence levels. Further research needs to target objective memory performance and subjective confidence using real-life scenes as tested with the present VR setup. The differential delay- and body-effects in the present study suggest that memory performance and confidence rely on distinct functional mechanisms [135], potentially consistent with the classical two-component model of episodic memory highlighting the distinction between familiarity and recollection, with only the second leading to changes in confidence [110].

### Outlook

The present VR methodology and the present behavioral findings will enable to study several key aspects of EM, including its subjective, behavioral and neural mechanisms and may benefit basic memory research in healthy participants, the understanding of memory disorders, and potentially provide therapeutic options. Concerning the involved brain mechanisms of BSC-EM interactions it will be important to describe how BSC related neural systems described for body ownership and self-identification [136,137], self-location and first-person perspective [39,138], as well as for temporal aspects of self-related processing [24,137] will interact with the well-described memory circuits in the medial temporal lobe. However, as the typical encoding of everyday life events is always associated with multisensory information involving the body (only few of which we tested here), more detailed investigations are necessary, involving BSC constraints [43]. Moreover, beyond bodily cues, the present setup also allows to control, auditory, visual cues, social cues and language material during encoding and retrieval and thus to test its effects on EM in in rich real-life like scenarios. The same techniques could also be extended and adapted to the investigation of amnesic patients. Future work may study in particular the links between autonoetic consciousness and BSC. Finally, there is a growing interest in VR for education (e.g. neurosurgery, firemen, pilots), cognitive behavioral therapy (e.g. treating phobias or post-traumatic stress disorder), and as pain treatments [139,140]. Future work is needed in order to explore the differences between VR and traditional studies in terms of learning and performance. Adaptations of the present setup will allow to personalize memory scenarios for a given memory patient and may be beneficial in memory rehabilitation [141,142].

## Supporting information

S1 Fig False alarms depend on number of items changed. EM performance (false alarms) is indicated in percentage + SEM. (**) P < 0.01; (*) P < 0.05. S1 Figure A. False Alarm versus Number of Items changed (i.e., 1 item, 2 items, 3 items) for immediate body condition; Figure B. Confidence Rate for False Alarm versus Number of Items changed (i.e., 1 item, 2 items, 3 items) for immediate no-body condition. Figure C. False Alarm versus Number of Items changed (i.e., 1 item, 2 items, 3 items) for delayed body condition; Figure D. Confidence Rate for False Alarm versus Number of Items changed (i.e., 1 item, 2 items, 3 items) for delayed no-body condition.
(TIFF)

S1 Data Raw data. This folder contains the raw data collected in the study.
(TXT)
