# Supplementary figures and images for: First-person view of one’s body in immersive virtual reality: Influence on episodic memory
Source: PLoS One. 2019 Mar 7;14(3):e0197763. doi: 10.1371/journal.pone.0197763 (PMC6405051; doi:10.1371/journal.pone.0197763)

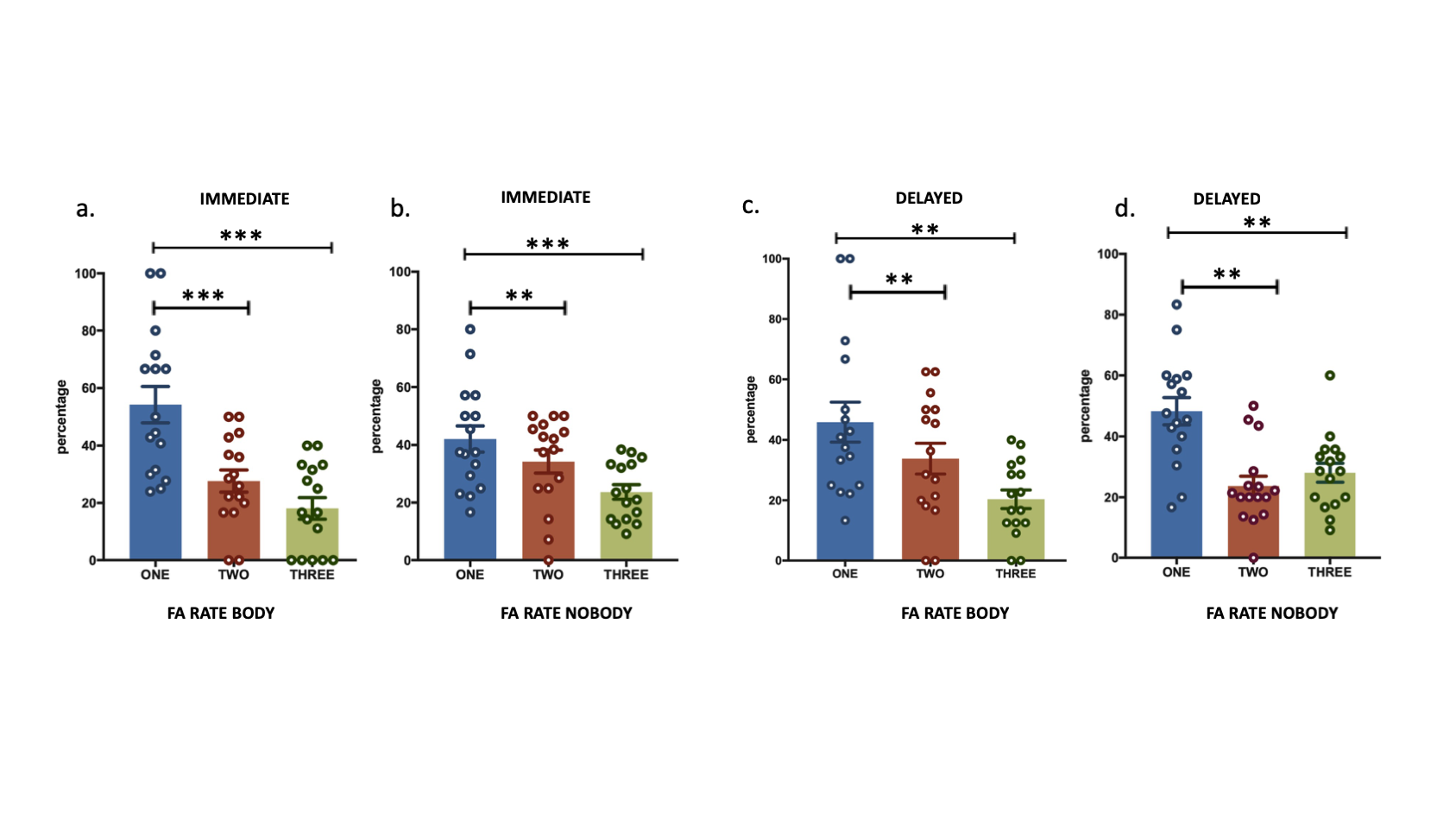

Supplement: S1 Fig — EM performance (false alarms) is indicated in percentage + SEM. (**) P < 0.01; (*) P < 0.05. S1 Figure A. False Alarm versus Number of Items changed (i.e., 1 item, 2 items, 3 items) for immediate body condition; Figure B. Confidence Rate for False Alarm versus Number of Items changed (i.e., 1 item, 2 items, 3 items) for immediate no-body condition. Figure C. False Alarm versus Number of Items changed (i.e., 1 item, 2 items, 3 items) for delayed body condition; Figure D. Confidence Rate for False Alarm versus Number of Items changed (i.e., 1 item, 2 items, 3 items) for delayed no-body condition. (TIFF) [file pone.0197763.s001.tiff]
